# Association Between Thygeson Superficial Punctate Keratitis and Celiac Disease

**DOI:** 10.7759/cureus.80252

**Published:** 2025-03-08

**Authors:** Adam Tagmouti, Hamza Lazaar, Meryem Benchekroun, Taha Boutaj, Saad Benchekroun, Abdellah Amazouzi, Lalla Ouafa Cherkaoui

**Affiliations:** 1 Ophthalmology A, Hopital des Specialités, Centre Hospitalier Universitaire Ibn Sina, Rabat, MAR

**Keywords:** ciclosporine a, cornea, dendritic, keratitis, thygeson

## Abstract

Thygeson’s superficial punctate keratitis (TSPK) is a rare, bilateral, chronic epithelial keratopathy characterized by recurrent exacerbations and remissions. Although its pathophysiology remains unclear, immunological and viral mechanisms have been implicated, with associations reported between TSPK and HLA-DR3. Similarly, celiac disease (CD) is an autoimmune disorder linked to HLA-DQ2 and HLA-DQ8, and HLA-DR3-DQ2. We report the case of a 20-year-old female with celiac disease who presented with TSPK. Clinical findings included stellate epithelial opacities and a pseudo-dendritic lesion, treated successfully with artificial tears, topical cyclosporine A, and therapeutic contact lenses. This case highlights a rare association between TSPK and CD, potentially explained by shared HLA genotypes, underscoring the need for further investigation into their immunogenetic link.

## Introduction

Thygeson’s superficial punctate keratitis (TSPK) is a rare bilateral, chronic inflammatory epithelial keratopathy characterized by recurrent episodes of exacerbations and remissions [1]. While its etiopathogenesis remains unclear, both viral and immunological mechanisms have been implicated as potential triggers. Notably, Darell RW reported a significant association between Thygeson’s keratitis and HLA-DR3 and DW3 [2]. 

Celiac disease (CD), on the other hand, is a chronic autoimmune disorder triggered by the ingestion of gluten in genetically predisposed individuals. It is defined by specific serological markers and histological findings. CD is strongly associated with HLA-DQ2 and HLA-DQ8 alleles, with the HLA haplotype DR3-DQ2 conferring a particularly high risk of disease development, especially during early childhood [3, 4].

We report the case of a 20-year-old female patient with a history of celiac disease who presented with Thygeson’s epithelial keratitis in its dendritic form and was treated with cyclosporin A.

## Case presentation

We report the case of a 20-year-old female patient with a 4-year history of celiac disease under a strict gluten-free diet with no other medical history, who presented with complaints of fluctuating visual blurriness, photobia, and foreign body sensation that had persisted intermittently for two years. The patient had previously undergone treatment with antibiotics and antiviral agents without any significant improvement.

**Figure 1:**
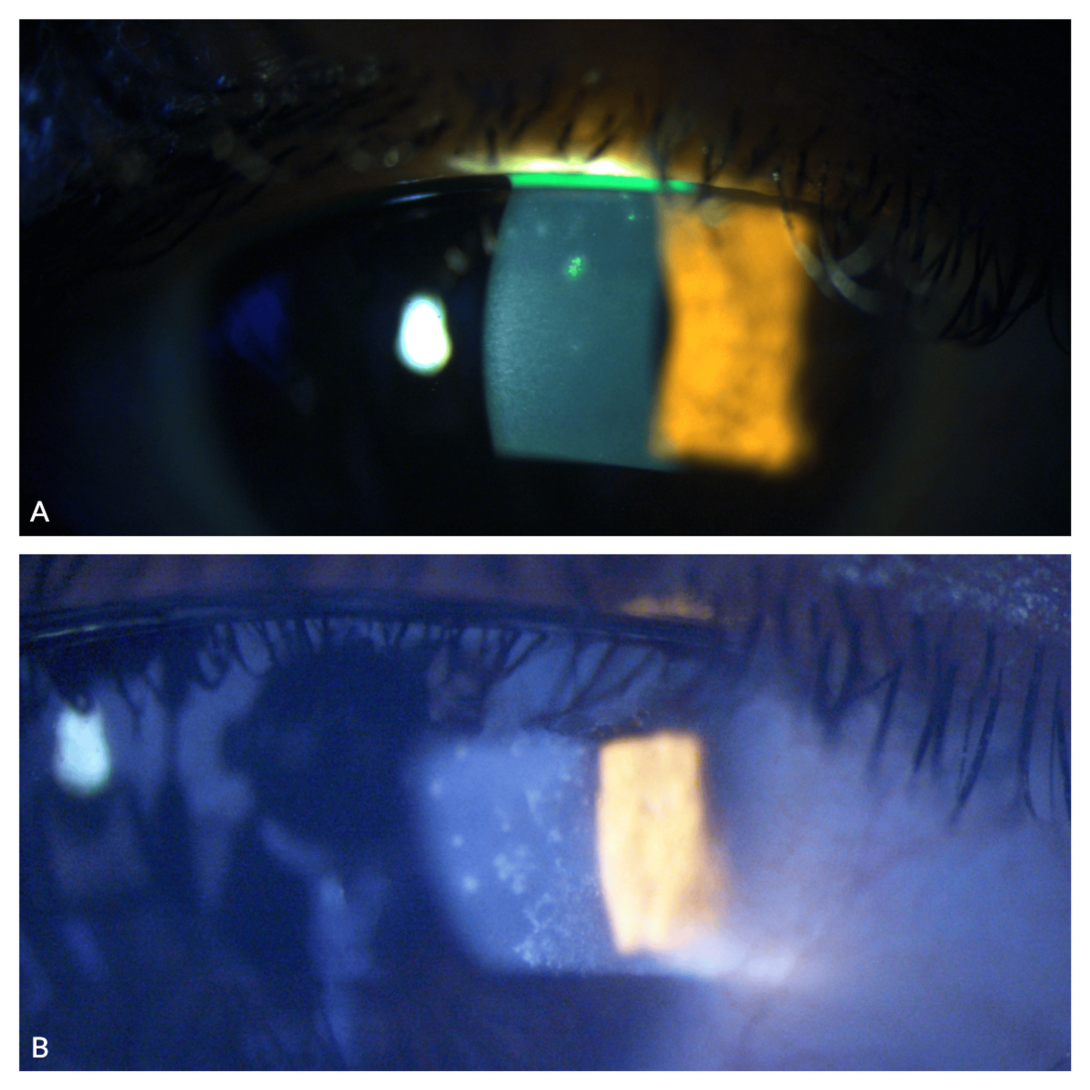
Slit-lamp examination of the right (A) and left eyes (B) showing whitish-grey epithelial lesions.

**Figure 2:**
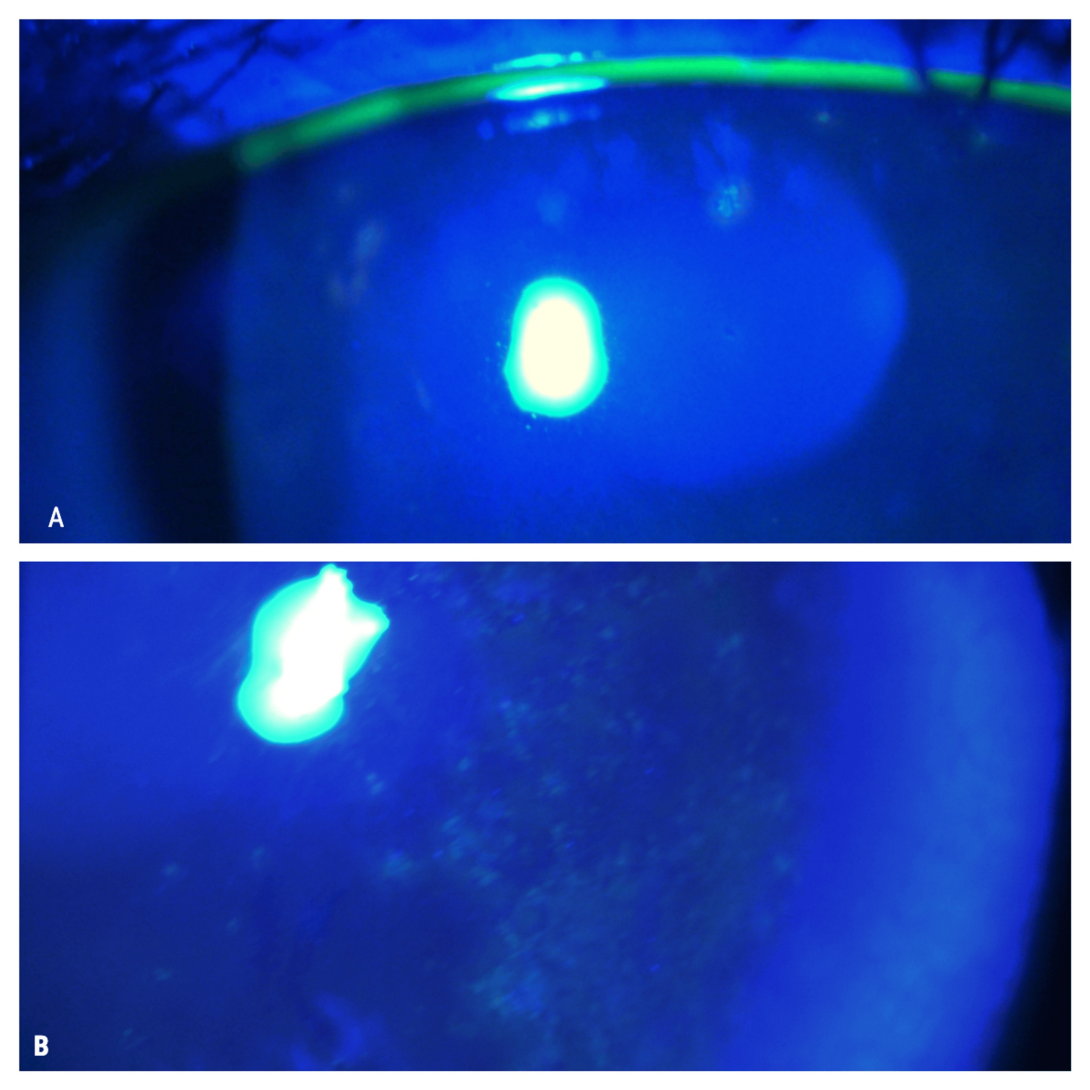
Slit-lamp examination of the right (A) and left eyes (B) showing staining of fluorescein of the epithelial lesions.

**Figure 3:**
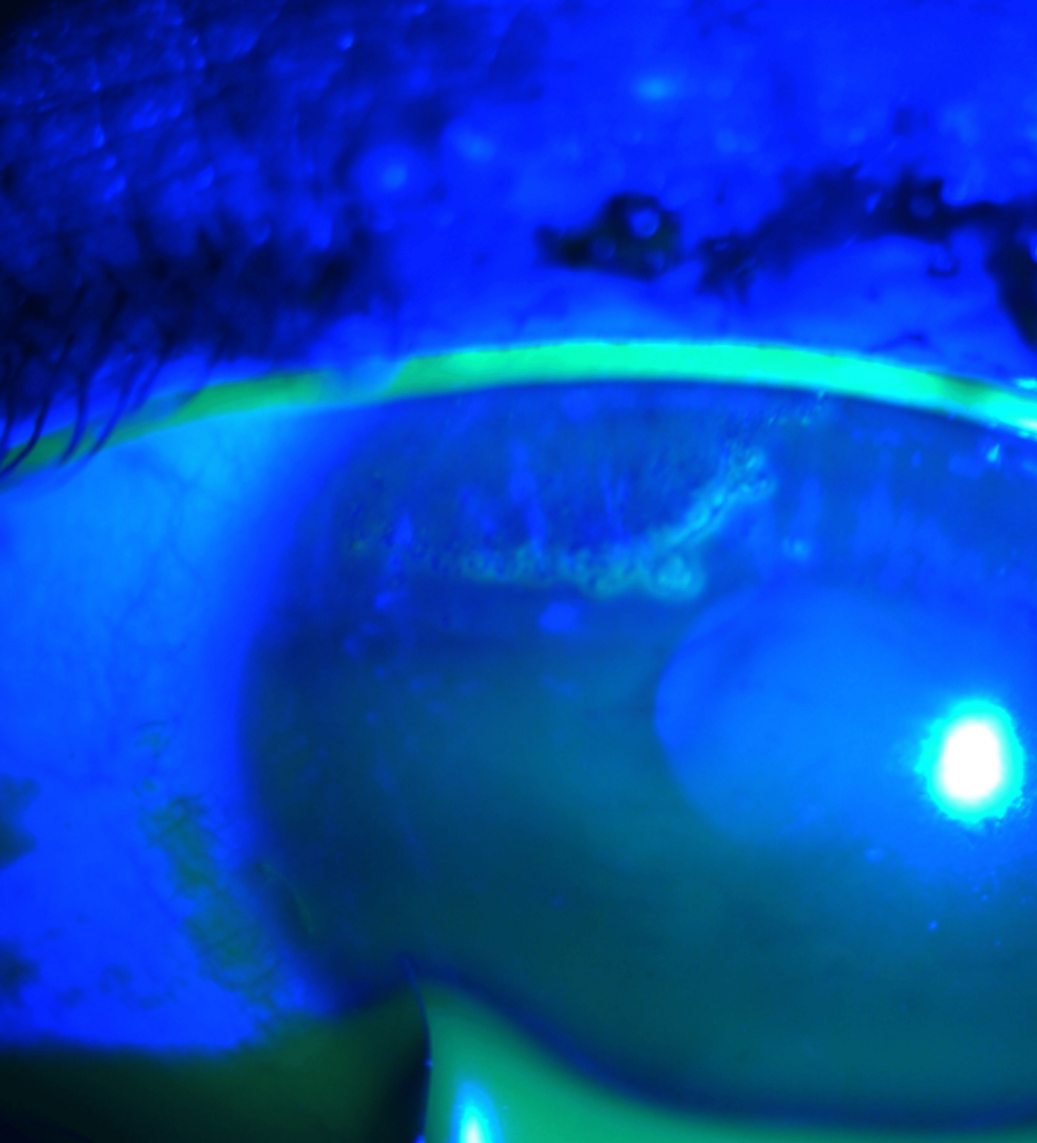
Slit-lamp examination showing dendritic lesion of the left eye and some superficial punctate keratitis.

Ophthalmological examination revealed a best-corrected visual acuity of 8/10 on the Snellen scale and an intraocular pressure of 13 mmHg in both eyes. Ocular motility was unremarkable. Slit-lamp examination in both eyes showed mild conjunctival hyperemia and numerous, slightly elevated, evanescent, stellate and grey-whitish epithelial opacities (Figure 1) that stained positively with fluorescein (Figure 2). Additionally, a pseudo-dendritic lesion was observed in the left eye (Figure 3). The corneal sensitivity was intact and equal in each eye according to a cotton swab test and the break-up time (BUT) was 10 seconds in both eyes.

The anterior chamber was quiet, and the lens was clear in both eyes. Fundus examination was unremarkable, with a disc of clear and well-defined margins and a good foveal reflex. An inflammatory workup including erythrocyte sedimentation rate (ESR) and C-reactive protein (CRP) returned negative, and no corneal sampling was performed.

Given the clinical findings, a diagnosis of Thygeson’s superficial punctate keratitis was established. The patient was treated with artificial tears (sodium hyaluronate 0.2%) and fluorometholone (0.1%) four times daily for a month, and topical cyclosporine A 2% eye drops to be administered three times daily, with a gradual tapering over a period of six months.

At the 2-month follow-up, the patient showed flat intraepithelial scars. They were requested to attend follow-up examinations in 1 month, 3 months, 6 months, and 12 months.

Clinical remission was achieved six months after the initiation of treatment, with the best-corrected visual acuity (BCVA) of 10/10. No recurrence of lesions observed over a three-year follow-up period.

## Discussion

Initially described in 1950 by Philip Thygeson [5], Thygeson’s keratitis is a rare recurrent, transient, bilateral keratopathy characterized by exacerbations and remissions that may last from months to decades [6]. TSPK affects both sexes equally, although some studies report a higher prevalence among females [5]. It can occur at any age, with a mean onset age of 30 years [7].

The pathophysiology of TSPK remains unclear, but viral and immunological mechanisms have been suggested. Viral infections such as Epstein-Barr Virus (EBV), varicella zoster virus (VZV), and herpes simplex virus (HSV) have been reported in the literature as potential triggers [8]. Darell et al. highlighted a significant association between TSPK and HLA-DR3 [2]. T-cell-mediated immune mechanisms likely contribute to the disease, as evidenced by the efficacy of immunosuppressive agents like cyclosporine A and tacrolimus in symptom management [9].

The most frequently reported symptoms of TSPK include foreign body sensation (48.8%), photophobia (41.9%), blurred vision (36.0%), and tearing (15.1%) in some studies [10]. The condition is almost always bilateral, with rare unilateral cases described [11]. The epithelial opacities are typically central, oval, or stellate, and white or gray, sparing the peripheral cornea. The dendritic pattern is rare and can mimic early herpetic keratitis [12].

Topical corticosteroids remain the mainstay of TSPK treatment, significantly improving the clinical picture by minimizing symptoms and reducing pain, though they may prolong the disease course [13]. Antibiotics are ineffective. Immunomodulatory agents have shown superior outcomes and fewer side effects compared to corticosteroids [14].

Epithelial debridement or chemical cauterisations are disapproved because of subsequent scarring and ulcerations [2].

This case is notable for three reasons: the rarity of TSPK, the dendritic presentation, and its association with celiac disease. CD is one of the most common autoimmune disorders, affecting approximately 1% of the general population [15]. It is strongly linked to HLA class II D-region markers, particularly DR3 and DQ2, with over 90% of patients carrying the DR3 allele [16]. The shared HLA-DR3 genotype between CD and TSPK suggests a possible immunogenetic link between these two conditions.

## Conclusions

This case highlights the rare association between Thygeson’s superficial punctate keratitis (TSPK) and celiac disease, both conditions potentially linked by shared HLA-DR3 genetic predisposition. The patient responded well to immunomodulatory treatment with topical cyclosporine A, achieving complete clinical remission within six months and showing no recurrence over three years of follow-up. This underscores the importance of considering autoimmune comorbidities in the management of atypical or refractory cases of TSPK and supports the role of immunomodulation as an effective therapeutic strategy. Ophthalmologists should consider screening for celiac disease in any case of Thygeson's keratitis, given the similar genetic predisposition. Further studies are needed to explore the immunogenetic relationship between TSPK and autoimmune diseases like celiac disease.
